# Histological incorporation of acellular dermal matrix in the failed superior capsule reconstruction of the shoulder

**DOI:** 10.1186/s40634-019-0189-1

**Published:** 2019-05-25

**Authors:** Matthew J. Ravenscroft, James A. Riley, Barnes W. Morgan, Dilraj S. Sandher, Saurabh S. Odak, Preethi Joseph

**Affiliations:** 1 Trauma and Orthopaedic Surgeon, Stepping Hill Hospital, Poplar Grove, Hazel Grove, Stockport, SK2 7JE UK; 2 Trauma and Orthopaedics Specialist Registrar, Stepping Hill Hospital, Poplar Grove, Hazel Grove, Stockport, SK2 7JE UK; 3 Histopathologist, Stepping Hill Hospital, Poplar Grove, Hazel Grove, Stockport, SK2 7JE UK

**Keywords:** Superior capsule reconstruction, Rotator cuff tear, Graft failure, Histology, Human acellular dermal allograft

## Abstract

**Background:**

Superior capsule reconstruction addresses massive rotator cuff tears using allografts and aims to restore the natural superior constraint of the shoulder and therefore shoulder biomechanics and function. There is no evidence relating to the histological incorporation of these grafts.

**Methods:**

27 superior capsule reconstructions were performed between June 2016 and November 2017. Follow-up was with clinical assessment and Magnetic Resonance Imaging, to identify graft failure. Reverse total shoulder replacement was offered for ruptured grafts and the graft was sent for histological analysis along with the footprint of graft attachment where possible.

**Results:**

Five patients (18.5%) had evidence of graft failure, three of whom (11.1%) underwent revision procedures. Of the five ruptures, four failed at the glenoid insertion, and one was an intra-substance tear. Histological analysis showed extensive fibroblastic infiltration. The intra-substance tear showed some vascularity at the medial and lateral ends, and one of the glenoid pull-outs demonstrated micro-calcification and osteoid formation. There was no evidence of in-growth into the bone.

**Discussion:**

An inflammatory response to the grafts was seen, with neo-vascularisation, and micro-calcification observed. These findings are from ruptured grafts, so may not represent the characteristics of those which have not ruptured. Further evidence from explanted intact grafts could be gained to improve our understanding of its incorporation.

**Level of evidence:**

Level IV evidence

## Introduction

Superior capsule reconstruction (SCR) is a novel technique described to address irreparable supra-spinatus or infra-spinatus tears (Hirahara & Adams, 2015; Burkhart et al., 2016). These tears have traditionally been very difficult to manage, especially in younger, more active patients, and treatment options include patch augmentation, partial repair, or reverse shoulder arthroplasty. Superior capsule reconstruction aims to restore the natural constraint to the superior translation seen with massive rotator cuff tears (Hirahara & Adams, 2015). Superior capsule reconstruction is indicated by massive cuff tears in the presence of a working deltoid muscle, and without any degenerative joint disease (Hirahara & Adams, 2015; Burkhart et al., 2016; Mihata et al., 2012).

Biomechanical studies have shown that patients with irreparable rotator cuff tears also have defects of the superior capsule (Mihata et al., 2016a). Whilst the rotator cuff prevents superior subluxation of the humeral head, the actual stability of the gleno-humeral joint superiorly comes from the capsule. Current evidence suggests that restoring the integrity of the superior capsule can help improve stability, and therefore functional outcomes, in massive rotator cuff tears (Mihata et al., 2012).

Superior capsule reconstruction can be performed either arthroscopically, or through an open procedure. It involves the use of either a human dermal allograft (Arthroflex, Arthrex. Naples FL.), a fascia lata autograft, or autologous hamstrings grafts, attaching to the superior glenoid and greater tuberosity, secured with bone anchors (Hirahara & Adams, 2015; Burkhart et al., 2016; Mihata et al., 2012; Mihata et al., 2016a; Petri et al., 2015).

Clinical studies have shown good short to mid-term outcomes following superior capsule reconstruction, with significant improvement in function and range of motion. Mihata et al. found that at a mean follow up of 34 months, 83.3% of 23 SCRs had no evidence of graft failure or cuff re-tear (Mihata et al., 2013). This is supported by Denard et al. who found a one year follow up graft failure rate of up to 18.6%, with most these failures occurring at the humeral side (Denard et al., 2018).

.A recent case study published showed failure at 6 months, with detachment of the graft from the glenoid (Zerr et al., 2017). The authors elected to repair this, re-attaching it and performing a side-to-side repair. They alluded to the possible cause of this rupture a being related to impingement under the acromion, leading to abrasion and graft rupture, but this would be expected to occur mid-substance or near the tuberosity insertion (Zerr et al., 2017). Biomechanical studies showed that when performed with acromioplasty, there was no superior migration of the humeral head, inferring no instability, and that there is less contact area, and therefore less abrasion of the graft (Mihata et al., 2016b).

Histological studies from acellular dermal matrix for SCR in rabbits has shown that there was fibroblastic infiltration, along with chronic inflammatory cells and eosinophils, which over time matured into tenoblasts and tenocytes (Kim et al., 2017). Microscopic analysis showed that over time, type I collagen fibres in the matrix took on the appearance of a mature tendon, with cellular repopulation, formation of neo-tendon, and incorporation into adjacent tendons. There was no evidence of neovascularisation. This suggests that the acellular dermal matrix acts as a scaffold for remodelling of the host tissue (Kim et al., 2017). There is no evidence with regards to the histological incorporation in human subjects, and it remains to be seen as to whether good histological incorporation is indeed a predictor of good clinical outcomes.

The authors aimed to find out what the fate of the acellular dermal matrix was after superior capsule reconstruction. In order to assess this, failed explanted grafts at revision surgery were sent for histological analysis, to test the hypothesis that the grafts caused a local inflammatory response, acting as a tissue scaffold.

## Methods

Three experienced Consultant Orthopaedic surgeons with a special interest in shoulder surgery performed the superior capsule reconstructions arthroscopically for large, irreparable rotator cuff tears confirmed on magnetic resonance imaging (MRI) scan. The large irreparable tears were those found to measure 30–50 mm in size on MRI scans. Oxford Shoulder Scores were performed pre-operatively. The procedures were all carried out under general anaesthesia with a regional block, in the beach-chair position. The superior glenoid was prepared with a radio-frequency wand and PowerPick drill (Arthrex, Naples Fl.), and the medial footprint was created to bleeding subchondral bone. The humeral head was debrided in a similar fashion to create the lateral footprint. Two 3 mm SutureTak (Arthrex, Naples Fl.) anchors were used medially on the glenoid surface, and two SwiveLock 4.75 mm anchors (Arthrex, Naples Fl.) were used laterally on the humeral head footprint. The area was then measured with calibrated arthroscopic probes to give the graft size. The standard 3.5 mm thick Arthroflex acellular dermal matrix was cut to the required size, leaving at least a 5 mm border around the anchors to reduce sutures cutting through, and at least 10 mm on the tuberosity side to cover the footprint. This was then rail-roaded along the medial sutures and tied in place, with a SpeedBridge (Arthrex Naples Fl.) created laterally and completed with a further two SwiveLock 4.75 mm anchors. The infra-spinatous was sutured to the posterior edge of the graft. In addition to this, a sub-acromial bursectomy was performed, and sub-scapularis was debrided. A long head of biceps tenotomy was not routinely performed.

Post-operatively, the patients were given a shoulder immobilising sling for six weeks, and were seen at ten days by an Extended Scope Physiotherapist to commence range of motion exercises. They underwent a standardised physiotherapy rehabilitation programme, with an emphasis on limiting external rotation for two months. They were seen by the Consultant Orthopaedic surgeon at three months post operatively for clinical assessment, and for MRI scanning. They were reviewed again at six months and one year, to review their clinical function, MRI, and patient related outcome scores using the Oxford shoulder score.

Patients were assessed for clinical and radiological signs of graft failure, and when evident, were offered revision surgery, to a reverse total shoulder replacement. Graft failure was confirmed in all with MRI scans, showing either loss of continuity in the substance of the graft, or anchor pullout from the footprint. Those electing to undergo revision had their grafts removed, and sent off for histological analysis. If there was any evidence of failure from the three-month appointment onwards, they were offered revision surgery.

During the revision procedure, the grafts were removed with the anchors and footprint of bone intact where possible, in order to assess graft incorporation at the footprint area. It was not possible to remove the grafts with a cuff of infra-spinatus left attached, so we were not able to assess integration at this aspect of the graft.

A Consultant histopathologist performed histological examination with sections taken along the long axis of the graft in the medial to lateral plane, in order to identify histological changes seen at the medial footprint, mid-substance and lateral footprint. They were prepared with haematoxylin and eosin staining and viewed under low and high power microscopes to assess their macroscopic and microscopic appearance. The aim was to identify any evidence of graft integration, for example, with micro-calcification of the graft, or neo-vascularisation.

## Results

Between June 2016 and November 2017, three surgeons in our centre had performed 27 superior capsule reconstructions, with a median patient age of 60 years, with more procedures being performed in females (63%). There were no significant differences between the patients with intact grafts and those with ruptures with respect to age, gender and cuff tear size. All patients underwent pre-operative MRI scanning, showing large rotator cuff tears (30–50 mm) without significant signs of osteoarthritis of the gleno-humeral joint. One procedure was performed to address a massive rotator cuff tear in the presence of an existing anatomical total shoulder replacement.

The mean Oxford shoulder score pre-operatively was 18.8, and at an average of six months post-operatively, it was 30.1, an improvement of 11.3. Five patients (18.5%) had radiological evidence of graft failure on MRI scans, all within six months from the procedure. Three patients (11.1%) decided to undergo revision procedures. The remaining two did not want a revision as they felt that functionally they did not require further intervention. The mean Oxford Shoulder Scores for the five patients with graft failure was 19.6 pre-operatively, and 29.3 post-operatively, an improvement of 9.7. This difference was not statistically significant to those with intact grafts (*p* = 0.5).

Of those grafts that showed evidence of failure, four (80%) had failed at the glenoid insertion, and one (20%) was an intra-substance tear. Of the three patients that underwent revision and graft analysis, one was the mid-substance tear, and the remaining two were glenoid side failure. When these two were removed, intra-operatively it was found that it was due to medial anchor pull out.

### Histology of mid-substance tear

Histological assessment of the graft showing mid-substance failure showed that macroscopically, there were no significant differences between the two sides. Microscopic assessment of the graft showed collagenous tissue with partial coverage by macrophages.

The areas immediately adjacent to the area of graft failure (lateral aspect of medial fragment and medial aspect of lateral fragment) showed acanthotic fragments of mainly non-viable tissue, with occasional fibroblastic activity. There was no significant evidence of inflammation or viable blood vessel formation.

The opposite ends of the grafts, around the footprints, showed chronic inflammatory cell infiltrates with giant cells and prominent vascularity. There was no evidence of bony in-growth or on-growth at either the humeral or glenoid aspect.

### Histology of glenoid sided failure

The first graft assessed was sent with samples of bone from the footprint area. This showed that there was scarring of the periosteum from the overlying graft, with fibro-fatty infiltrates. At the interface of the graft and bone there was evidence of fibroblasts and aggregates of multi-nucleate giant cells, but no suggestion of incorporation into the bone itself, or in-growth into the graft.

**Fig. 1:**
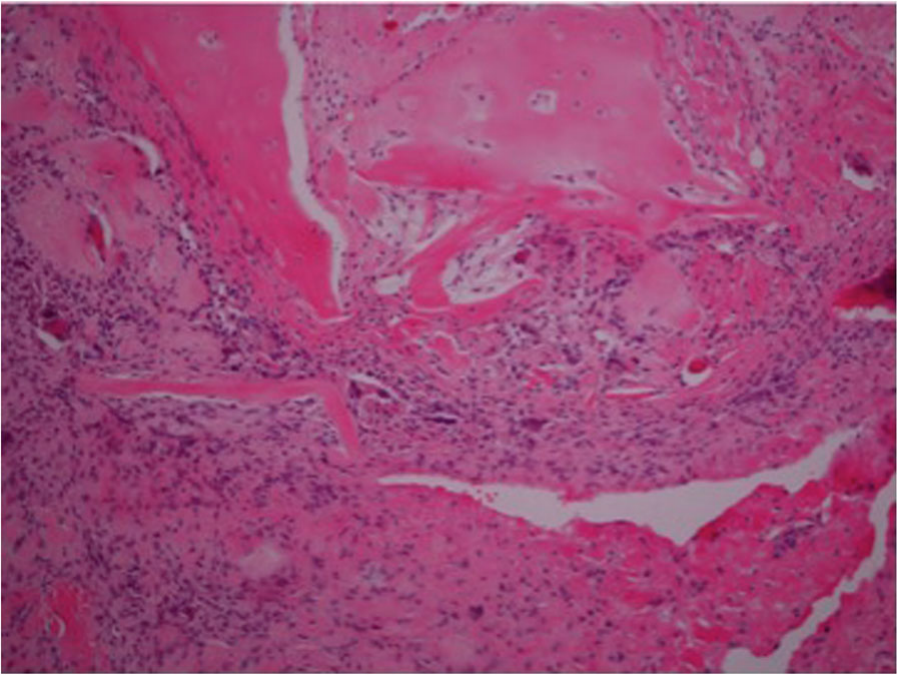
High-power (40x) microscopic appearance of medial aspect of the graft, with extensive inflammatory cell infiltrates (purple nucleated cells)

**Fig. 2:**
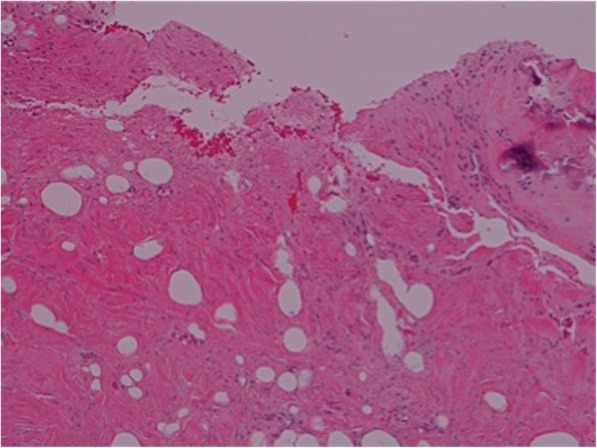
High-power (40x) microscopic appearance of lateral aspect of the graft with multi-nucleated osteoblastic type cells

The second graft underwent analysis of the medial, middle and lateral sections. The microscopic appearance of the lateral aspect of the graft (Fig. 1.) showed scanty evidence of calcified bony spicules and clusters of osteoclast type giant cells. The sample from the medial part (Fig. 2.) showed more cellular activity at the periphery, with evidence of fibroblastic proliferation. There was also evidence of bony osteoid formation, micro-calcification and chronic inflammation. This suggests there was some evidence of bony in-growth into the graft at both the glenoid and humeral aspects.

None of the three grafts demonstrated any evidence of infection or malignancy.

## Discussion

The graft rupture rate seen was 18.5%, which is very similar to that demonstrated in the literature, with a revision rate of 11.1%, although more recent evidence indicates a failure rate as low as 4% (Mihata et al., 2018). The majority of grafts that failed did so at the glenoid side. This is in contrast to the current evidence, as Denard demonstrates that most grafts they studied failed at the humeral insertion (Denard et al., 2018).

The histological samples showed that the acellular dermal matrix caused a significant inflammatory response, demonstrated by the presence of fibroblasts. At the footprints of one of the grafts the graft, there was evidence of bony integration, in-growth, which is highlighted by osteoclastic activity, microcalcification, and the evidence of bony spicules. One of the graft samples even showed prominent vascularity, indicating good integration of the acellular dermal matrix (Aurora et al., 2007).

There was evidence that the graft stimulated an inflammatory response to the bone at glenoid the footprint, with giant cell and fibroblast proliferation (Bertasi et al., 2017). There was not, however, any evidence that the grafts studied had shown signs of bony on-growth. This finding could help explain a possible reason for graft failure, as it was found that 80% failed at the glenoid side. If the graft is not incorporating into the bone, this could be the reason why the two grafts removed had pulled out with their SutureTak anchors from the glenoid footprint. Macroscopically and microscopically, the graft-anchor interface was intact, suggesting this was not the site of failure, but it is more likely to be anchor failure.

Clinically, the improvement in Oxford Shoulder Scores performed pre-operatively and at six months post-operatively was not statistically significant for the group with intact grafts compared to those with a ruptured graft. This yet again appears to conflict with the current literature, as Denard et al. showed a 55% reduction in patient satisfaction when the graft had not completely healed (Denard et al., 2018). In our population, two patients with ruptured grafts were satisfied enough with their function that they elected not to undergo revision procedures.

Previous evidence from Zerr et al. implicated abrasion on the graft as a possible reason for failure, and advocated acromioplasty to reduce this (Zerr et al., 2017). Our findings do not support this conclusion, as they hypothesised acromial abrasion would cause mid-substance tears, but the majority of our graft failures were at the glenoid attachment with the anchors. In addition, all cases had previously had, or underwent, acromioplasty, and there was still an 18.5% rate of graft ruptures.

The evidence from rabbits shows that the complete grafts had similar findings, with fibroblast, chronic inflammatory cell and eosinophilic infiltration. They did not find any evidence of neovascularisation, and hypothesised that the graft acts as a scaffold (Kim et al., 2017). Our results support this, but take the evidence further, as they are samples taken from humans, in vivo, and did show evidence of capillary infiltration.

These results are from ruptured grafts, so it is important to take into account the fact that they may not be representative of the findings in successful grafts. The fact that there are three samples with multiple sections from each does give a good indication of the cellular interaction on and around the graft. It could be argued that the fact that they ruptured could mean they have a different histological fate compared to those grafts that do not, but the fact that sections from different parts of several grafts show good bony integration and capillary infiltration hopefully makes these comparable to those grafts, which did not rupture.

In order to improve the evidence base, histological samples from non-ruptured grafts would be needed, to assess their fate once implanted. The limitation of this, would be the removal of the graft in an otherwise well functioning reconstruction. This is a relatively new procedure, and as these patients progress, they may develop some degenerative joint disease, requiring reverse arthroplasty. It is at this point that the intact grafts could be removed, and histological assessment performed to address this issue.

## Conclusion

The histological evidence from three revised superior capsule reconstructions indicates that the acellular dermal matrix samples studied produced a local inflammatory response when implanted into the shoulder. There was extensive inflammatory cellular infiltration seen, and even evidence of bony and vascular infiltration into the graft at the peripheries, which indicates that the graft is a good biological scaffold. None of the grafts showed evidence of on-growth onto the bone at the graft footprint.

Evidence from 27 superior capsule reconstructions performed by three surgeons over a year and a half show promising results. There was a mean improvement in Oxford shoulder score of 11.3 comparing pre-operative scores with scores at an average of six months post-operative. There was a graft rupture rate of 18.5% and a revision rate of 11.1%, which is in line with the existing data.
